# Omission of non-poly(A) viral transcripts from the tissue level atlas of the healthy human virome

**DOI:** 10.1186/s12915-020-00907-z

**Published:** 2020-11-25

**Authors:** Signe Altmäe, Nerea M. Molina, Alberto Sola-Leyva

**Affiliations:** 1 Department of Biochemistry and Molecular Biology, Faculty of Sciences, University of Granada, 18071 Granada, Spain; 2 Instituto de Investigación Biosanitaria ibs.GRANADA, 18014 Granada, Spain; 3 Competence Centre on Health Technologies, 50411 Tartu, Estonia

**Keywords:** GTEx, Poly(A) tail, RNA-seq, Virus, Virome

## Abstract

A recent paper in *BMC Biology* entitled “A tissue level atlas of the healthy human virome” by Kumata et al. describes a meta-transcriptomic analysis of RNA-sequencing datasets from the Genotype-Tissue Expression (GTEx) Project. Using a workflow that maps the GTEx sequences to the human genome, then screens unmapped sequences to detect viral transcripts, the authors present a quantitative analysis of the presence of different viruses in the non-diseased tissues of over 500 individuals and assess the impact of these viruses on host gene expression. Here we draw attention to an issue not acknowledged in this study. Namely, by relying solely on GTEx datasets, which are enriched for transcripts with poly(A) tails, the analysis will have missed non-poly(A) viral transcripts, rendering this tissue level atlas of the virome incomplete.

A commentary on Kumata et al. (BMC Biol 18:55, 2020).

Viruses are obligate parasites and require a living cell to complete their life cycles. Like mRNAs in the eukaryotic host cell, RNAs of many DNA and RNA viruses generate polyadenylated transcripts (i.e., transcripts containing 3′ poly(A) tails) that are synthesized post-transcriptionally [1], and in some RNA viruses also by direct transcription from poly(U) sequence on the stretched template strand [2, 3]. The viral poly(A) tails are important for regulating RNA stability and translation initiation, mimicking roles of the stable poly(A) tails in eukaryotic mRNA [4].

Many viruses, however, generate transcripts without poly(A) tails, a feature that has been maintained over evolution, especially in positive-strand RNA viruses as for instance are dengue virus, West Nile virus, Japanese encephalitis virus, yellow fever virus, Zika virus, bovine viral diarrhea virus, and hepatitis C virus in the *Flaviviridae* family [4–6]. Other important examples of non-poly(A) viral RNA transcripts are adenovirus-encoded non-coding RNA viral-associated RNAs and herpesvirus EBV-encoded non-coding small RNAs (EBERs) (the gold standard clinic markers for detection of EBV latent infection in specimens) [7]. Viral-encoded non-poly(A) RNAs have an important role in different physiological conditions and illnesses, including viral life cycle and function, and host cell immune evasion and transformation [8].

Next-generation sequencing offers high sensitivity, specificity, and reproducibility in detection of low levels of transcripts thereby serving as a sensitive and reliable tool to qualify and quantify viruses at DNA and RNA levels [9]. Nevertheless, depending on the exact sequencing protocol of choice, the non-polyadenylated viral RNA sequences could be detected or discarded (Fig. 1). The recent *BMC Biology* article by Kumata et al. presented the first tissue level atlas of the human virome by analyzing the RNA-seq data from the GTEx database [10]. GTEx uses oligo (dT) primers for obtaining poly(A)-enriched fraction in the initial RNA purification step, meaning that only the RNA transcripts with poly(A) tail will be enriched and sequenced [11]. We believe that Kumata et al. study has overlooked this important aspect, and although the first comprehensive investigation of the human virome in somatic tissues was presented, an important part of the human virome was not detected. A recently published study comparing poly(A)-enriched RNA-seq and non-poly(A)-selected RNA-seq in the lung virome analysis from the same samples supports our concern, as in this study it was demonstrated clearly that poly(A)-enriched RNA-seq failed to detect several viruses [7]. Furthermore, Kumata et al. conclude that mainly DNA viruses shape the healthy human virome as most of the detected viruses in their study were DNA viruses, although they acknowledge the possibility that the detection sensitivity of RNA viruses could have been lower [10]. Indeed, especially RNA viruses lack poly(A) tail [5, 6], which could be one solid explanation why RNA viruses were under-detected and DNA viruses predominated in the study by Kumata et al.

**Fig. 1:**
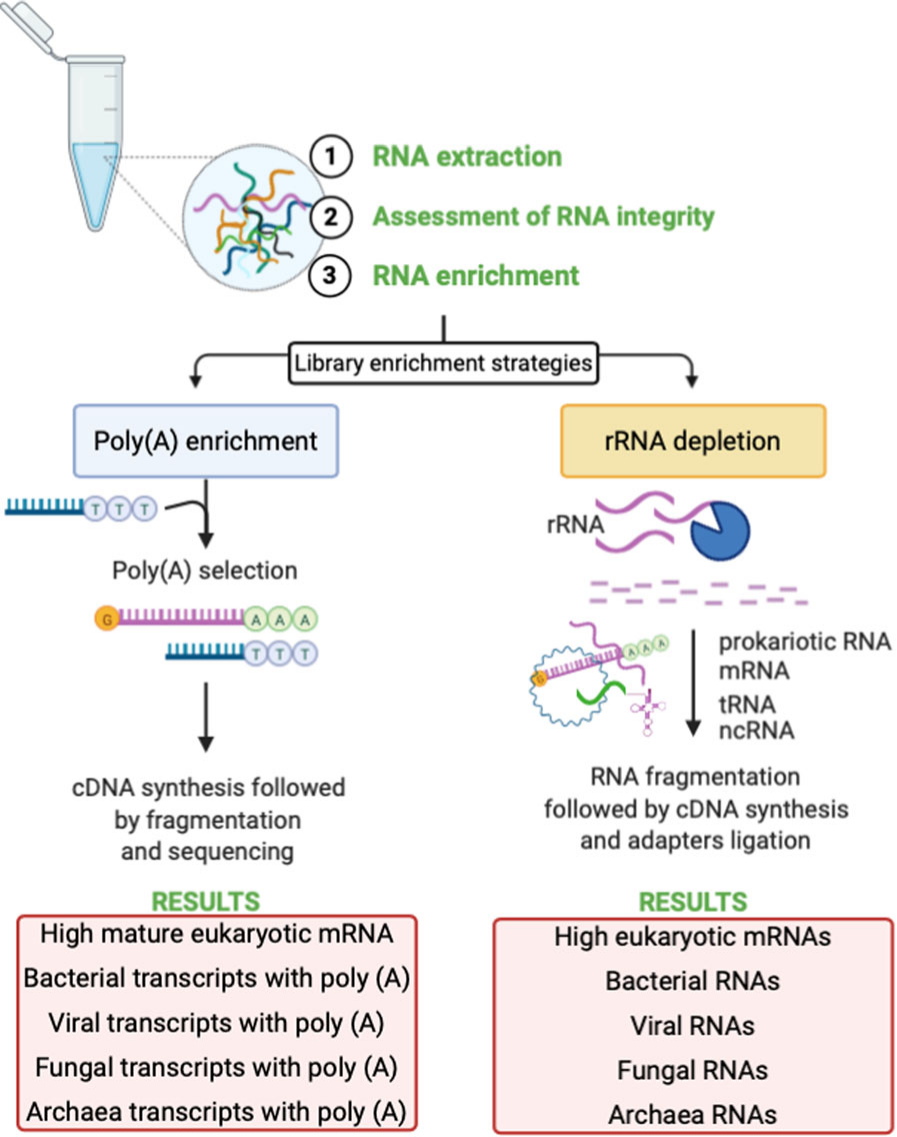
. Simplified illustration of the two main protocols for analyzing RNA-seq

Before other researchers are motivated to apply their meta-transcriptomic study approach [10] to other datasets with the aim of revealing the impact of viral infections on human health, we would like to highlight that the choice of sequencing protocol is crucial in obtaining and interpreting the study findings. In short, the recently presented tissue level atlas of the healthy human virome should be acknowledged as a partial tissue level atlas, and the comprehensive investigation should be completed with meta-transcriptome analysis of data generated using the total RNA extraction method in order to achieve a more complete view of the human virome.

## Data Availability

Not applicable.

## Abbreviations

EBERs: EBV-encoded non-coding small RNAs
EBV: Epstein-Barr virus
mRNA: Messenger RNA
