# Supplementary material for: Omission of non-poly(A) viral transcripts from the tissue level atlas of the healthy human virome
Source: BMC Biol. 2020 Nov 25;18:179. doi: 10.1186/s12915-020-00907-z (PMC7690156; doi:10.1186/s12915-020-00907-z)
Supplement: Related texts

# Reply: Response to “Omission of non-poly(A) viral transcripts from the tissue level atlas of the healthy human virome”

**Authors:** Ryuichi Kumata, Jumpei Ito, Kei Sato

**Affiliations:** grid.26999.3d 0000 0001 2151 536X Division of Systems Virology, Department of Infectious Disease Control, International Research Center for Infectious Diseases, Institute of Medical Science, The University of Tokyo, 4-6-1 Shirokanedai, Minato-ku, Tokyo, 1088639 Japan

Correspondence: ksato@ims.u-tokyo.ac.jp

In our recent paper in *BMC Biology* titled “A tissue level atlas of the healthy human virome” [10], we performed meta-transcriptomic analysis using the RNA-sequencing (RNA-Seq) dataset from the Genotype-Tissue Expression (GTEx) Project, which includes 8991 RNA-Seq data obtained from 51 somatic tissues from 547 individuals. In this study, we detected 39 viral speciesOmission of non-poly(A) viral transcripts from the tissue in at least one tissue and furthermore investigated associations between virus infection (e.g., hepatitis C virus and some human herpes viruses) and human gene expression [e.g., type I interferons (IFNs) and IFN-stimulated genes].

As described in the first sentence of the “Method” section of our paper [10], we used the pair-ended, poly(A)-enriched RNA-Seq data provided by GTEx (version 7.p2). Altmäe et al. are correct to point out that because the dataset we used lacks the information of non-poly(A) RNAs, our study does not include the data of non-poly(A) viral transcripts. In this regard, some RNA viruses (e.g., Flaviviruses) potentially produce non-poly(A) RNAs. Therefore, in terms of the description of the human virome, particularly that of human RNA viruses, we cannot exclude the possibility that the results shown in our recent paper [10] are incomplete. If there were large datasets available that included the data of non-poly(A) RNAs, it would be possible to survey the bona fide human virome more efficiently and effectively, as suggested by Altmäe et al.

Some aspects of the technical limitations of our study were described in the “Discussion” and “Conclusion” sections of our study [10], and it was an oversight not to have also acknowledged the limitation of the available dataset. The GTEx project does not provide the dataset of non-poly(A) RNA-Seq and is not just specified for the investigation of the human virome. However, to our knowledge, the GTEx project provides public access to the biggest set of transcriptome data for non-diseased human tissues (i.e., 8991 RNA-Seq data obtained from 51 somatic tissues from 547 individuals) that exists to date. Using this dataset, we quantified viral “mRNA” transcripts [i.e., poly(A)-added viral RNAs], and to the best of our knowledge, this is the first and biggest investigation addressing the presence of viruses in a variety of human tissues. We recognize, however, that our meta-analysis shows the human tissue virome based on the data of viral “mRNA” transcripts and does not include non-poly(A)-added RNAs. The technology of next-generation sequence data analysis progresses rapidly, and the publicly available datasets are increasing day by day. The advanced investigation of the human virome using non-poly(A) RNA-Seq data is an intriguing prospect, and we look forward to seeing the results of such an endeavor in the future.
